# Validity of social–emotional screening tool for newborns and infants: The effects of gender, ethnicity and age

**DOI:** 10.3389/fpsyg.2022.960086

**Published:** 2022-10-19

**Authors:** Faye Antoniou, Ghadah S. Al-Khadim

**Affiliations:** ^1^Department of Educational Studies, National and Kapodistrian University of Athens, Athens, Greece; ^2^Department of Psychology, College of Arts, Taif University, Taif, Saudi Arabia

**Keywords:** social–emotional skills, infants, mean differences, BPSC, measurement invariance, construct validity

## Abstract

The purpose of the present study was to test the measurement invariance of the baby pediatric symptom checklist (BPSC) across gender and age as a means to provide for valid comparisons in point estimates across groups. A secondary goal involved confirming the earlier identified factor structure and re-examining the presence of differentially item functioning in the BPSC across grouping variables. Participants were 601 children (322 boys and 279 girls) aged below 1 year (*n* = 294) and 1 year to 12 months (*n* = 307). Data were collected as part of the National Health Interview Survey in 2020. Results related to gender indicated satisfying all five levels of measurement and structural invariance. When testing measurement invariance across age groups, a few modifications were necessary. Specifically, partial metric invariance was satisfied after freeing slope estimates of items 1 and 2, partial scalar after freeing intercept terms of items 6 and 10, and partial residual invariance through freeing error terms of items 2 and 12. These non-invariant estimates, however, provide support for partial invariance and do not invalidate the comparison of latent means. The BPSC can be used for the valid assessment of social–emotional skills in infants up to the age of 18 months.

## Introduction

Based on UNICEF, in 2020, 2.4 million newborns died in the first month of life around the globe. This estimate represents approximately 30 deaths per 1,000 live births. The number of newborn deaths has increased by 40% in 2020 compared to 1990. In the United States, the rate is approximately 5 deaths per 1,000 live births although this estimate varies widely by race and ethnicity. Benova et al. (2020) emphasized the need to identify behavioral and growth indicators that are valid predictors of a newborn’s health as a means to strengthen the quality of measurable predictors and outcomes. Valid measurement of social emotional difficulties can lead to early intervention and treatment especially for disorders prevalent to infants such as internalizing and externalizing problems, such as temper tantrums. Interestingly, the validity of measurement is likely compromised across domains. For example, Williams et al. (2018) reported that broad developmental measures likely miss young children’s social and emotional needs.

Social–emotional difficulties in infancy have had important consequences for future development and adaptation as well as success in school (Bolten, 2013). Problem areas have been predominantly identified in sleep (Mindell et al., 2017), feeding (Hemmi et al., 2011), and crying (Alvarez, 2004), all three identified as important predictors of subsequent behavioral problems (Bolten, 2013; Sidor et al., 2013). Behavioral problems have been associated with impaired parent–child relationships (Papousek and von Hofacker, 1998) and school problems (Hyde et al., 2012). Specifically, sleep problems have been predictive of poor social and emotional functioning and the presence of psychiatric problems (Berger et al., 2012; Winsper and Wolke, 2014; Sadeh et al., 2015).

McIntosh et al. (2021) in a review of the literature pointed to the fact that most screening measures of socio-emotional screening targeted children 18 months and older pointing to the scarcity of measures for newborns. Further concerns about the measurement of social and emotional skills at a very young age pointed to the lack of rigorous psychometric data on the validity of the available measures (Pontoppidan et al. (2017). To this end, the Baby Pediatric Symptom Checklist (Sheldrick et al., 2013) was developed to screen for social and emotional symptoms in newborns and infants from birth to 18 months and has provided evidence in favor of construct and convergent validity, reliability, and bias (via Differential Item Functioning) across gender, parent education, and family income. Despite the rigorous methodology employed and the positive results in favor of the baby pediatric symptom checklist (BPSC), additional tests of validity are warranted before the measure can be utilized in tests of mean-level differences across gender, ethnic, and age groups within the range of 0–18 months.

There is ample evidence that gender differences are present in temperament during infancy with also large effect sizes (e.g., in effort control favoring girls, see Else-Quest et al., 2006). Some studies have reported that girls have higher levels in positive affect (LaFrance et al., 2003) and fear (Olino et al., 2013), which are also accompanied by lower activity levels compared to boys (Eaton and Enns, 1986), although meta-analytic findings concluded small overall effect sizes. Furthermore, differences in the variability in measuring temperament were reported with boys having more variable temperament estimates compared to girls (Else-Quest et al., 2006). This rather inconclusive evidence suggests the need to investigate such effects further, as there are concerns that they are linked to methodological shortcomings such as the source of data collection (parent report vs. observational studies), or across parent’s gender (Seifer et al., 1994).

Hispanic and Latino groups are the largest and fastest-growing populations in the U.S. (Federal Interagency Forum on Child and Family Statistics, 2017) with also unique characteristics and challenges. For example, one-third (30%) of Latino children live in poverty, and more than 60% in households with low incomes (Murphey et al., 2014). Developmental findings have also indicated lower scores in Hispanic infants on both cognitive and social outcomes (Borkowski et al., 2002) and also salient differences in rearing practices, and socio-economic factors (Moore et al., 1997) suggesting they are 3–4 times at a greater risk for developmental delays, language and social–emotional difficulties (Jahromi et al., 2012).

### Importance, novelty, and goals of the present study

The well-being of newborns and babies lies on the monitoring of specific crucial indicators. Tracking growth is then dependent upon the validity, importance, and relevance of these growth behaviors and indicators (Larson and Mercer, 2004). The necessity of identifying those indicators goes without saying as social and emotional problems have been strongly linked to adaptation and school success in later life (Kagan et al., 1999), the presence of psychopathological tendencies (Else-Quest et al., 2006), and adjustment in later life (Rothbart et al., 2001).

The purpose of the present study was to test the validity of the BPSC, a freely available measure for the assessment of social/emotional well-being in children under the age of 18 months (see Supplementary material). Specifically, we sought to confirm the sample structure of the BPSC using a nationally representative U.S. sample. Furthermore, we attempt to test requisite steps before conducting mean level tests across gender and age groups, that is, the presence of measurement and structural invariance (Meredith, 1993), verify the reported simple structure (replicate the factor solution reported by Sheldrick et al., 2013), and examine additional indices of dimensionality and internal consistency reliability.

## Materials and methods

### Participants and procedures

Data came from the National Health Interview Survey which provides detailed data on the health of the U.S. nation since 1957 via structured household interviews. The present paper utilized the data from 2020, which included revisions to some of the earlier surveys. There were 601 children (*N*_boys_ = 322, *N*_girls_ = 279) aged below 1 year (*n* = 294) or 1 year to 12 months (*n* = 307). This age classification comprised the grouping variable in our invariance modeling. Furthermore there were 142 Hispanic children (23.6%) and 459 non-Hispanic (76.4%).

### Measures

The BPSC was developed as part of the Survey of Wellbeing of Young Children and in its final form included 12 items (Sheldrick et al., 2013) that are appropriate for the measurement of social–emotional skills in newborns and infants below the age of 18 months. The scaling system included 3 options from “not at all,” to “somewhat” to “very much.” In its final form, it included the measurement of 3 domains, namely, (a), inflexibility (b), irritability, and, (c) difficulty with routines, derived from the conceptual frameworks of Rothbart (1981). Internal consistency reliability was assessed using the omega coefficient (Raykov, 1997) which is most appropriate for non-tau-equivalent instruments. Results indicated adequate levels of internal consistency reliability across domains (Omega_Inflexibility_ = 0.894; Omega_Irritability_ = 0.898; Omega_Difficulty with Routines_ = 0.827).

### Data analyses

Data were analyzed using structural equation modeling and specifically the confirmatory factor analysis framework with ordinal indicators. Furthermore, a measurement and structural invariance protocol was employed to verify the equivalence of form and function across gender and age groups as described below. First, the configural model was tested which evaluated the equivalence in form across gender and age groups. This was followed by the metric model in which the relationships between items and latent variables were tested. The third model, which represents a prerequisite before testing for mean differences was the scalar model specifying, in addition to the configural and metric models, the equivalence of intercept terms. Further constraints included “strict” invariance, that is, the equivalence of item residual variances, followed by tests of the equivalence of variances in the latent factors. All analyses were conducted using Mplus 8.8 (Muthen and Mythen, 1998–2018). Models were nested as they included the same items and latent constructs but were differentiated from each other by specifying additional restrictions (e.g., equivalence of slopes, thresholds, etc.). Consequently, Chi-square difference tests were utilized when contrasting models.

## Results

### Construct validity of the BPSC

Table 1 displays model fit statistics of the BPSC *via* three competing models. Model 1 (M1) is a fixed slope 3-factor correlated model, resembling the Rasch-model idea of fixed discrimination parameters. Model 2 (M2) estimates 3-correlated factors with slopes freely estimated and Model 3 (M3) is a bifactor model with items loading on both a general factor and also specific factors. Using inferential statistical criteria results indicated that the free-slopes model (M2) was superior to the fixed slopes model using a loglikelihood difference test which is distributed as a Chi-square statistic [*χ*^2^(12) = 235.057, *p* < 0.001]. The more parameterized bifactor model was associated with a slightly better model fit, which by no means justified the estimation of the additional 9 parameters [*χ*^2^(9) = 11.133, *p* = 0.267]. Consequently, due to parsimony, the optimal model fit was attained using a three-factor correlated model (M2) as in the original Sheldrick et al. (2013) study. This model had all descriptive fit indices [i.e., comparative fit index (CFI), Tucker–Lewis index) over 0.95, and unstandardized residual values less than 5%, being indicative of “exact model fit.” Thus, the construct validity of the BPSC was replicated with the present data. Further tests of measurement and structural invariance utilized M2.

**Table 1:** Model fit of BPSC using item factor analysis.

Model	LL	Npar	scf	Model comparison	LRTS*	dtsc	sLRTS*	df	Value of p^**^
M1. Fixed slopes	−3,601.52	27	0.991	–	–	–	–	–	–
M2. Free slopes	−3,474.83	39	1.018	M1 vs. M2	253.386	1.078	235.057	12	<0.001
M3. Bifactor	−3,469.17	48	1.018	M2 vs. M3	11.314	1.016	11.133	9	0.267

LL, Loglikelihood; Npar, number of estimated parameters; scf, scaling correction factor; LRTS, −2(LL0−LL1); DTSC, differences between two scaling correction factors: dtsc (*p*0 × *c*0 − *p*1 × *c*1)/(*p*0 − *p*1); df, difference degrees of freedom, *p*1−*p*0; sLRTS, LRTS/dtsc.

** The value of *p* reflects a Chi-square statistic of the sLRTS for the respective degrees of freedom d.f.

### Measurement invariance across gender

Measurement and structural invariance across gender are shown in Table 2. Initially, a configural model was fit to the data, which showed an acceptable model fit in that the 3-factor correlated simple structure fit the data well for both boys and girls. Further testing involved more constrained models as a means to examine the decrease in fit due to non-invariance. The metric model specified the equivalence of factor loadings across gender with thresholds being freely estimated. Results indicated that constraining slopes to be equivalent in boys and girls was not associated with significant decrements in model fit [Difftest(9) = 14.202, *p* = 0.115]. The scalar model involved testing the equivalence of thresholds across boys and girls in addition to the equivalence of slopes. The scalar model was also supportive of these constraints by not being linked to a significantly worse model fit [Difftest(21) = 21.372, *p* = 0.436]. The results from the first three models pointed to the presence of full measurement invariance and justify the proper comparison of latent means across gender. The fourth layer of constraining involved the equivalence of residual estimates across groups, namely testing “strict” invariance. As shown in the table, constraining error terms to be equivalent across groups was again supported [Difftest(12) = 17.789, *p* = 0.122]. Consequently, the amount t of unexplained variance at the item level was equivalent across groups. Further tests involved testing for structural invariance, namely the invariance of factor variances. Using the difftest procedure results indicated equal variances across latent variables for both boys and girls [Difftest(3) = 1.227, *p* = 0.747]. Model fit was excellent with CFI = 0.978, and root mean squared error of approximation (RMSEA) being at 3.7%.

**Table 2:** Model fit testing measurement and structural invariance across gender, age and ethnic groups.

Model tested	Chi-square	*χ*^2^ df	Value of *p*	CFI	TLI	RMSEA	RMSEA low CI	RMSEA high CI	RMSEA value of *p*
Gender									
M1a. Configural Model	153.828	102	<0.001	0.974	0.966	0.041	0.027	0.054	0.866
M2a. Metric Model	164.385	111	<0.001	0.973	0.968	0.040	0.026	0.052	0.903
M3a. Scalar Model	185.135	132	0.002	0.973	0.973	0.037	0.023	0.048	0.970
M4a. Residual Invariance	185.135	132	0.002	0.973	0.973	0.037	0.023	0.048	0.970
M5a. Factor Variance Invariance	178.097	135	0.008	0.978	0.978	0.033	0.018	0.045	0.992
Ethnicity									
M1a. Configural Model	152.999	102	<0.001	0.972	0.964	0.041	0.027	0.054	0.875
M2a. Metric Model	151.999	111	0.007	0.978	0.974	0.035	0.019	0.048	0.974
M3a. Scalar Model	181.482	132	0.003	0.973	0.973	0.035	0.021	0.047	0.979
M3b. Partial Scalar Model (free threshold of item 8)	174.996	131	0.006	0.976	0.976	0.033	0.019	0.046	0.988
M4a. Residual Invariance	174.996	131	0.006	0.976	0.976	0.033	0.019	0.046	0.988
M5a. Factor Variance Invariance	177.406	134	0.007	0.976	0.977	0.033	0.018	0.045	0.991
Age groups									
M1a. Configural Model	139.344	102	0.008	0.979	0.973	0.035	0.018	0.049	0.966
M2a. Metric Model	164.367	111	0.001	0.970	0.965	0.040	0.026	0.052	0.903
M2b. Partial Metric Model (free slopes of items 1–2)	151.418	109	0.005	0.976	0.971	0.036	0.021	0.049	0.962
M3a. Scalar Model	188.182	132	0.001	0.969	0.969	0.038	0.024	0.049	0.959
M3b. Partial Scalar Model (free thresholds of items 6,10)	177.637	128	0.002	0.972	0.971	0.036	0.022	0.048	0.973
M4a. Residual Invariance	188.182	132	0.001	0.969	0.969	0.038	0.024	0.049	0.959
M4b. Residual (free error variances of items 2 and 12)	160.748	126	0.020	0.981	0.980	0.030	0.013	0.043	0.995
M5a. Factor Variance Invariance Model	167.820	129	0.012	0.978	0.978	0.032	0.016	0.044	0.993

In model 3b, the first threshold of item 6 and the second threshold of item 10 were left free to vary across groups. The Chi-square test reflects the omnibus statistical test of the model; *χ*^2^ df = The Chi-square degrees of freedom; value of *p* = observed probability; CFI, Comparative fit index; TLI, Tucker–Lewis index; RMSEA, Root mean squared error of approximation; CI, Confidence intervals at the 95% level, lower and upper; RMSEA value of *p* = observed probability that the RMSEA is different from zero. A null effect points to insufficient evidence that unstandardized residuals are not zero, thus, zero is a plausible estimate.

### Measurement invariance across ethnic groups

Measurement and structural invariance across Hispanic and Non-Hispanic groups are shown in Table 2. The configural model was again acceptable supporting the 3-factor simple structure. The metric model also verified the equivalence of slopes linking the items to the latent factors [Difftest(9) = 6.080, *p* = 0.732]. The scalar model suggested non-equivalence of the threshold of item 8 [Difftest(21) = 35.627, *p* = 0.024], which was left free to vary across ethnic groups pointing to the presence of partial scalar invariance [Difftest(21) = 26.929, *p* = 0.137]. “Strict invariance” or testing the equivalence of residual variance estimates across groups was also supported [Difftest(12) = 14.213, *p* = 0.287]. Last, tests of the variability in the latent variables between ethnic groups also led to a conclusion of invariance [Difftest(3) = 4.413, *p* = 0.220]. Model fit was excellent across all models.

### Measurement invariance across age

Tests of measurement and structural invariance were conducted across newborns up to 12 months old and between 1 year and 18 months old. The same analytical protocol described above was utilized here as well. As shown in Table 2, the configural model was again associated with excellent model fit (i.e., CFI = 0.979, RMSEA = 0.035). Constraining item slopes on the three domains were associated with significant decrements in model fit [Difftest(9) = 23.258, *p* = 0.006]. Using modification indices results indicated that the slopes of items 1 and 2 needed to be freed. This partial metric invariance model (M2b) was associated with non-significant decrements in comparison to the configural model [Difftest(7) = 12.747, *p* = 0.079]. Constraining the thresholds across age groups was associated with significantly lower model fit [Difftest(21) = 39.223, *p* = 0.009] pointing to the need to free the thresholds of items 6 (0 vs. 1 and 2) and item 10 (0 and 1 vs. 2). After allowing these parameters to vary across age groups (M3b) results indicated achieving partial scalar invariance [Difftest(19) = 29.173, *p* = 0.063]. Further tests of the equivalence of residual variances pointed to discrepant estimates between the two groups [Difftest(12) = 37.340, *p* < 0.001]. The modification indices suggested significant decrements in the Chi-square if the residual variances of items 2 and 12 were freed (M4b). This suggestion was substantiated by the data [Difftest(10) = 16.741, *p* = 0.080]. The last model testing structural invariance suggested the equivalence of variability in the three latent constructs. Results pointed to non-significant decrements in model fit from adopting this assumption [Difftest(3) = 6.006, *p* = 0.111]. The current results suggested that the BPSC under these modifications would provide proper comparisons in the latent means across newborns (0–12 months) and infants (12–18 months).

### Latent mean comparisons across gender, ethnic groups, and age

**Figure 1:**
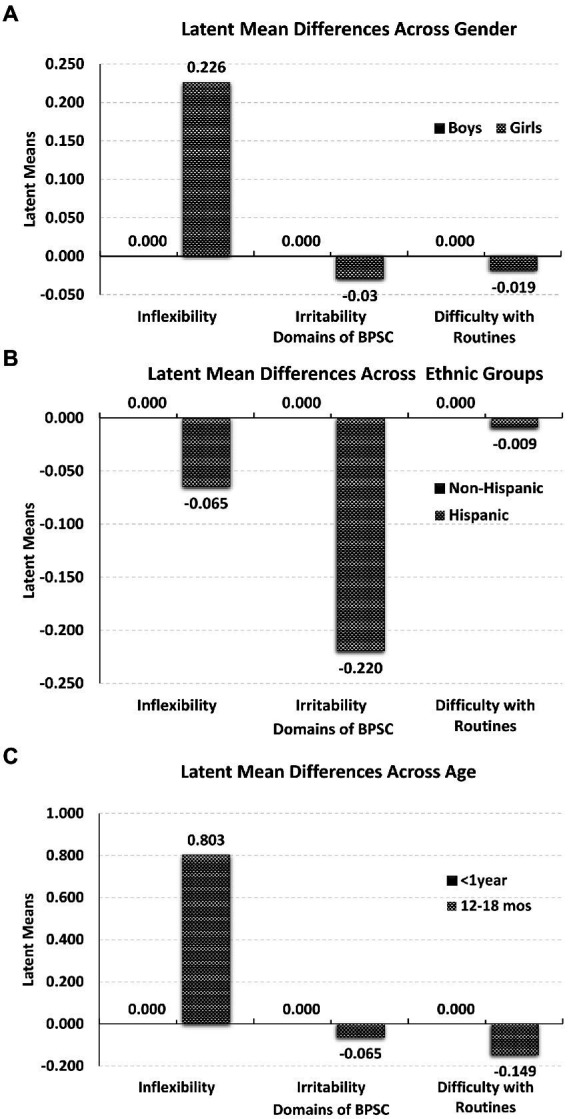
Latent mean differences across gender (upper panel-**A**), ethnic groups (middle panel-**B**), and age groups (lower panel-**C**) on the BPSC domains.

Following satisfying scalar (for gender groups) or partial scalar invariance (for ethnic and age groups) comparisons between means can be conducted. These comparisons at the latent level involve fixing the latent factor means of the reference group to zero and freely estimating the latent means of the comparison group. Results when comparing boys with girls indicated no significant differences in the latent means of *irritability* (*M*_girls_ = −0.030, *z* = −0.269, *p* = 0.788), and in *difficulty with routines* (*M*_girls_ = −0.019, *z* = −0.169, *p* = 0.866). The means of girls, however, was significantly higher compared to the mean of boys on inflexibility (*M*_girls_ = 0.226, *z* = 2.047, *p* = 0.041; Figure 1, upper panel), albeit with a small effect size.

When testing latent mean differences in the three domains of the BPSC across Hispanic and Non-Hispanic groups results indicated non-significant differences across all domains. Thus, there were no differences in *inflexibility* (*M*_Hispanic_ = −0.065, *z* = −0.931, *p* = 0.352), *irritability* (*M*_Hispanic_ = −0.220, *z* = −1.597, *p* = 0.110) or in *difficulty with routines* (*M*_Hispanic_ = −0.009, *z* = −0.065, *p =* 0.948, see Figure 1, middle panel).

Last, tests of latent mean differences in the three domains of the BPSC across age groups pointed to non-significant differences in *irritability* (*M*_12-18mos_ = −0.065, *z* = −0.557, *p* = 0.577), and *difficulty with routines* (*M*_12–18mos_ = −0.149, *z* = −1.274, *p* = 0.203). Differences were revealed in inflexibility, with infants (12 to 18 months old) having significantly higher means compared to newborns (*M*_12–18mos_ = 0.803, *z* = 6.456, *p* < 0.001, see Figure 1, lower panel).

## Discussion

The present study evaluated the psychometric properties of the BPSC as a means to provide valid assessments of latent mean differences across gender and age groups. Results favored the original simple structure of Sheldrick et al. (2013) and verified the measurement and structural invariance of the 3-factor solution across gender and age groups. Furthermore, indices of internal consistency reliability were exceptional pointing to minimal amounts of measurement error. Thus, the three facets of the instrument can be used for the valid assessment of temperament in infancy (Buss and Plomin, 1975).

Differences in latent means were revealed across gender with females having significantly higher difficulties in the inflexibility domain, albeit with a small effect size. This finding agrees with the results of Spinner et al. (2018) but disagrees with the earlier meta-analytic findings of Else-Quest et al. (2006) who reported no differences across gender in adaptability (*d* = −0.003, *p* = n.s.). Thus, the present findings add to the equivocal research findings from past studies. Another difference with the Else-Quest study was that the amount of variance of boys was significantly elevated compared to the respective estimate for girls in their meta-analytic study. In the present study, satisfying factor variance invariance (Model 5) suggested no differences in the variability of the latent construct of inflexibility/adaptability. However, one needs to weigh the magnitude of effect, which was small in the present study, as a function of relatively excessive levels of power, thus, there may not be a salient difference between the present and the Else-Quest et al. (2006) study’s findings. For age, older infants had significantly elevated scores on inflexibility and this finding agrees with the study by Rothbart and Mauro (1990) who reported a correlation of 0.57 across scores on adaptability and an age grouping variable (6-month-old vs. 12-month-old). Furthermore, the lack of finding significant differences in temperament across ethnic groups disagrees with earlier findings pointing to delays in Hispanic toddlers in social and emotional development (Borkowski et al., 2002).

In the future additional evidence of the validity of the BPSC can be attempted through testing longitudinal invariance, which is very important as newborns and infants age. Also, the invariance across races and different SES groups may further increase our understanding of mean differences across populations. Further tests can engage advances in psychometrics such as approximate measurement invariance (AMI) and the alignment method (Muthen and Asparouhov, 2018) in case partial measurement invariance fails so that latent mean comparisons can still be conducted.

## Data availability statement

Publicly available datasets were analyzed in this study. This data can be found at: https://www.cdc.gov/nchs/nhis/2020nhis.htm.

## Author contributions

FA conceptualized the study and contributed to data analyses and the write-up of the manuscript. GA-K contributed to data analyses and the write-up of the quantitative sections. Both authors approved the final draft of the manuscript. All authors contributed to the article and approved the submitted version.

## Funding

This project was funded by Taif University Researchers Supporting Project number (TURSP-2020/334), Taif University, Taif, Saudi Arabia.

## Conflict of interest

The authors declare that the research was conducted in the absence of any commercial or financial relationships that could be construed as a potential conflict of interest.

## Publisher’s note

All claims expressed in this article are solely those of the authors and do not necessarily represent those of their affiliated organizations, or those of the publisher, the editors and the reviewers. Any product that may be evaluated in this article, or claim that may be made by its manufacturer, is not guaranteed or endorsed by the publisher.

## Supplementary material

The Supplementary material for this article can be found online at: https: //www.frontiersin.org/articles/10.3389/fpsyg.2022.960086/full#supplementary-material
