# Supplementary material for: Validity of social–emotional screening tool for newborns and infants: The effects of gender, ethnicity and age
Source: Front Psychol. 2022 Oct 19;13:960086. doi: 10.3389/fpsyg.2022.960086 (PMC9627284; doi:10.3389/fpsyg.2022.960086)
Supplement: Supplementary file 1 [file Table_1.DOCX]

**Supplementary Material**

**Content and Rating Scale of BPSC**

| **Items BPSC** | **Not at all** | **Somewhat** | **Very Much** |  |
| --- | --- | --- | --- | --- |
|  | **Inflexibility** | | |  |
| 1.Does your child have a hard time being with new people? | 0 | 1 | 2 |  |
| 2.Does your child have a hard time in new places? | 0 | 1 | 2 |  |
| 3.Does your child have a hard time with change? | 0 | 1 | 2 |  |
| 4.Does your child mind being held by other people? | 0 | 1 | 2 |  |
|  | **Irritability** | | | |
| 5.Does your child cry a lot? | 0 | 1 | 2 |  |
| 6.Does your child have a hard time calming down? | 0 | 1 | 2 |  |
| 7.Is your child fussy or irritable? | 0 | 1 | 2 |  |
| 8.Is it hard to comfort your child? | 0 | 1 | 2 |  |
|  | **Difficulty with Routines** | | | |
| 9.Is it hard to keep your child on a schedule or routine? | 0 | 1 | 2 |  |
| 10.Is it hard to put your child to sleep? | 0 | 1 | 2 |  |
| 11.Is it hard to get enough sleep because of your child? | 0 | 1 | 2 |  |
| 12.Does your child have trouble staying asleep? | 0 | 1 | 2 |  |
